# Comparison of success rates in eye drop instillation between sitting position and supine position

**DOI:** 10.1371/journal.pone.0204363

**Published:** 2018-09-20

**Authors:** Tomoko Naito, Keiji Yoshikawa, Koji Namiguchi, Shiro Mizoue, Atsushi Shiraishi, Yuko Ichikawa, Miyuki Fujiwara, Takako Miki, Ryoichi Araki, Yuzo Umeda, Yuki Morizane, Fumio Shiraga

**Affiliations:** 1 Department of Ophthalmology, Okayama University Medical School Hospital, Okayama, Japan; 2 Yoshikawa Eye Clinic, Tokyo, Japan; 3 Department of Ophthalmology, Ehime University Graduate School of Medicine, Ehime, Japan; 4 Department of Gastroenterological Surgery, Okayama University Medical School Hospital, Okayama, Japan; National Eye Institute, UNITED STATES

## Abstract

**Purpose:**

To compare the success rates of eye drop instillation in the sitting position and supine position among Japanese patients with ocular diseases (cataract, glaucoma, or retinal and vitreous diseases).

**Methods:**

Patients who were hospitalized in Okayama University Hospital for eye surgery were studied. Instillation procedures of each patient in both the sitting and supine positions were recorded using a video camera at the time of instillation. We defined “success” when one drop fell accurately onto the ocular surface at the first attempt. Instillation of two or more drops, drops delivered to a site other than the eye surface, and touching the eyelashes, eyelids, or conjunctiva with the tip of the eye drop bottle were regarded as “failure”. We excluded patients with vision below counting finger.

**Results:**

One-hundred and two patients (54 males and 58 females, aged 70.2 ± 12.3 years) with ocular disease who were hospitalized for surgery (cataract: 61.8%, glaucoma: 15.7%, retinal and vitreous diseases: 22.5%) were included in this prospective observational study. The mean duration of eye drop use was 3.1 ± 5.2 years. The success rate of eye drop instillation was significantly higher in the supine position than in the sitting position (64.7% vs. 50%, *P* = 0.0039). The mean age was significantly higher in the failure group than in the success group (74.0 ± 11.5 vs. 67.7 ± 12.4 years, *P* = 0.0085) for the sitting position, but not significantly different for the supine position (72.3 ± 12.9 vs. 70.1 ± 12.0 years, *P* = 0.3849). No significant differences in mean duration of drop use, mean corrected VA, and mean spherical equivalent refraction were observed between success and failure groups, for both sitting and supine positions.

**Conclusions:**

In the present study, the success rate of eye drop instillation was significantly higher when applied in the supine position than in the sitting position.

## Introduction

Poor adherence to treatment is a well recognized problem. Proper administration of medicine is as important as proper choice of medicine for successful treatment, since the benefit from the treatment depends on correct administration. According to the World Health Organization (WHO) [1–2], the factors influencing adherence to medications can be aggregated into five dimensions: (1) patient-related factors (no subjective symptom or lack of patient’s understanding to disease condition), (2) therapy-related factors (discomfort or side effects), (3) condition-related factors (cognitive/affective status of the patient, medication regimen, severity of the disease, or difficulty of eye drop technique), (4) health system-related factors, and (5) social/economic factors. In ophthalmic treatment, the eye drop instillation technique is an important condition-related factors.

However, even experienced eye drop users struggle with successful instillation of glaucoma drops. In a study involving direct observation, although 93% of experienced drop users reported no problems instilling eye drops, only 31% were able to correctly instill an eye drop [3]. The common failures in eye drop instillation observed in glaucoma patients include the inability to instill a drop into the eye, instilling more than one drop, and touching the eye or periocular tissues with the bottle tip [3–8]. In our previous study of 78 glaucoma patients, 61.5% of the patients failed to instill one drop accurately onto the ocular surface at the first attempt [9]. The inherent difficulties of properly instilling an eye drop have been reviewed in various studies [10–11], one of which showed that only 39% of patients with glaucoma administered the drops successfully.

Adherence to ophthalmic treatment presents a particularly complex issue for elderly patients. Although eye drop bottles and ophthalmic compliance aids have been developed, they are still not effective enough to overcome the barrier regarding adherence to ophthalmic treatment at present. It is vital to consider reasons for nonadherence and provide individualized support to help overcome the treatment failure. Support for correct eye drop instillation technique is often overlooked, although there are educational support for treatment regimens, patient concerns, and consideration of ophthalmic compliance aids.

In the present study, we conducted a study comparing the success rate of eye drop instillation in the sitting and in supine positions among Japanese patients with ocular diseases, and examined whether change in posture alters the success rate.

## Patients and methods

A prospective observational study was conducted to assess the success rate of eye drop instillation in the sitting and supine positions by patients who were hospitalized at the Department of Ophthalmology of Okayama University Medical School Hospital for surgery because of cataract, glaucoma, or retinal and vitreous diseases.

### Participants

The study was conducted from January 2016 to January 2017. Patients who met all of the following inclusion criteria were eligible to participate in the study: (1) aged 20 years or older with a diagnosis of cataract, glaucoma, or retinal and vitreous diseases, and (2) hospitalized for surgery at the Department of Ophthalmology of Okayama University Medical School Hospital. Exclusion criteria were (1) a history of active ocular inflammation such as recurrent uveitis, scleritis, or corneal herpes, and (2) ocular injuries, intraocular surgery, or laser surgery within 3 months before participating in the present study.

### Methods

Participants were asked to demonstrate how they usually instilled eye drops using a 5-mL plastic bottle containing preoperative ophthalmic anti-bacterial solution (Cravit Ophthalmic Solution 0.5%, Santen, Japan) while they were in a sitting position and also in a supine position. The “sitting position” was defined as sitting on a chair or bed, and the “supine position” was defined as firmly lying on a hard bed. Each subject instilled the eye drop both in a sitting position and a supine position. The order of the two instillations was decided by the subject him/herself. One eye was selected per patient for this study. No instruction was provided to participants, and their instillation technique was recorded by examiners. The examiners (K.N. and T.N.) recorded the following performance of each participant at a distance of 0.5 meter from the eye: (1) taking the bottle and opening the cap by hand, (2) bringing the bottle above an eye, and (3) squeezing the bottle and instilling solution onto the ocular surface. A digital video recorder (HDR-XR520V, Sony, Japan) at high-resolution mode of 1,920 x 1,080/60p was used for recording the whole process of instillation.

### Criteria for judging success or failure of eye drop instillation

Two examiners (S.M. and Y.K.) who were not involved in digital video recording watched the recorded videos to judge the success or failure of eye drop instillation. The definition of success in eye drop instillation was when one drop was accurately instilled onto the ocular surface at the first attempt. The following situations were considered to be failure: (1) two or more drops were delivered in one attempt, (2) one drop was delivered to sites other than the eye surface, and (3) the tip of the bottle touched the eyelashes or bulbar conjunctiva.

The research protocol complied with the principles of the Helsinki Declaration and was approved by the Institutional Ethics Review Board (ERB) of Okayama University Medical School Hospital (Registration number: 1606-011-004). Written informed consent was obtained from all participants after a thorough explanation of this study objective and methods.

### Statistical analysis

Statistical analysis was performed using JMP software version 8.0 (SAS Institute Inc., Cary, NC, USA). Data analysis was performed independently at the Department of Gastroenterological Surgery, Okayama University. The mean ± standard deviation was used in the descriptive statistics of normally distributed data. A McNemar’s test was used to compare the difference between paired proportions. A *t*-test was used to compare normally distributed continuous variables between independent groups. A chi-square test was used to compare binomial proportions. A *P* value less than 0.05 was considered statistically significant.

## Results

### Demographic and clinical characteristics

The characteristics of the participants are shown in Table 1. A total of 102 subjects (44 males, 58 females) who fulfilled all criteria were enrolled. The mean age was 70.2 ± 12.3 years. Sixty-three patients underwent surgery for cataract (61.8%), 16 for glaucoma (15.7%), and 23 for retinal and vitreous diseases (22.5%). The mean corrected visual acuity (VA) was 0.84 ± 0.55 logMAR, mean spherical equivalent refraction was -1.18 ± 3.04 D, and mean duration of eye drop use was 3.1 ± 5.2 years.

**Table 1:** Characteristics of the study participants.

	Participants (n = 102)
**Age (years)**	70.2 ± 12.3 [22–91]
**Gender (n)**	
Male	44 (43.1%)
Female	58 (56.7%)
**Reasons for hospitalization (n)**	
Cataract	63 (61.8%)
Glaucoma	16 (15.7%)
Retinal and vitreous diseases	23 (22.5%)
**Corrected VA (logMAR)**	0.84 ± 0.55 [0–2.3]
**Spherical equivalent refraction (D)**	-1.18 ± 3.04 [-9.45–4.5]
**Duration of eye drop use (years)**	3.1 ± 5.2 [0–20]

### Success rates in sitting and supine position

**Fig 1:**
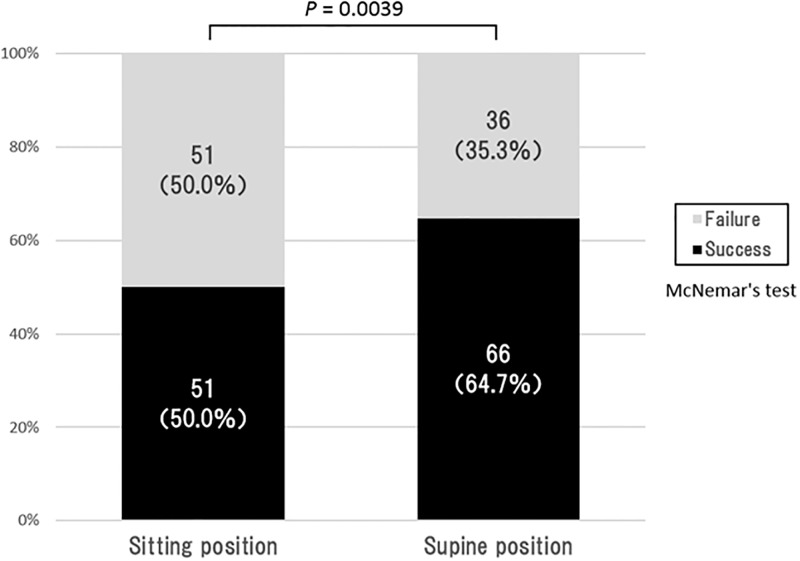
Comparison of eye drop success rates in sitting and supine positions. Data are expressed as n (%).

The results of comparison of the success rate of eye drop instillation in the sitting and supine positions are shown in Fig 1. The success rate was significantly higher in the supine position (64.7%) than in the sitting position (50%) (*P* = 0.0039; McNemar’s test). Forty-five (44.1%) of 102 patients were successful in both positions, 6 (5.9%) were successful only in the sitting position, 21 (20.6%) were successful only in the supine position, and 30 (29.4%) failed in the both positions. In addition, Tables 2 and 3 show the success rates of eye drop instillation in sitting position and supine position, when subjects were stratified into age groups. There was a significant decrease in success rate as age increased in the sitting position (p = 0.0006; Cochran-Armitage trend test), but there was no significant difference among age groups in the supine position (p = 0.0969; Cochran-Armitage trend test).

**Table 2:** Success rates of eye drop instillation in sitting position, stratified by age group.

Age group (years, number of subjects in parenthesis)	Success (no. of subjects)	Failure (no. of subjects)	Success rate
<60 (n = 13)	10	3	76.9%
60~69 (n = 27)	17	10	63.0%
70~79 (n = 40)	18	22	45.0%
>79 (n = 22)	6	16	27.3%

**Table 3:** Success rates of eye drop instillation in supine position, stratified by age group.

Age group (years, number of subjects in parenthesis)	Success (no. of subjects)	Failure (no. of subjects)	Success rate
<60 (n = 13)	11	2	84.6%
60~69 (n = 27)	17	10	63.0%
70~79 (n = 40)	25	15	62.5%
>79 (n = 22)	13	9	59.1%

### Mean ages in failure and success groups in sitting and supine positions

**Fig 2:**
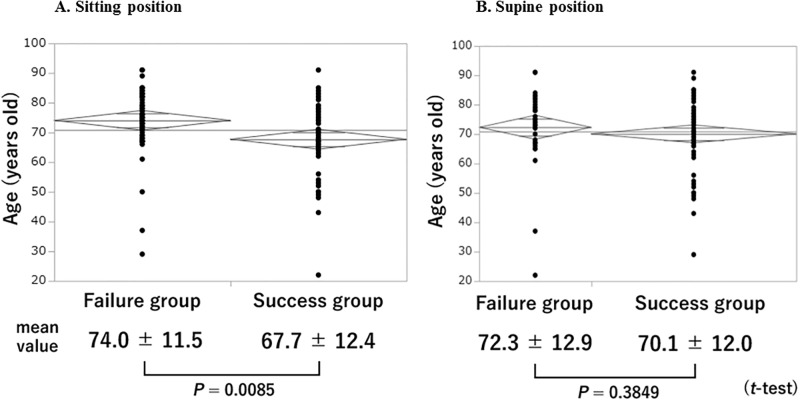
Comparison of mean age between the failure and success groups for sitting and supine positions. The horizontal line across the graph represents the mean of all subjects. Each diamond plot shows the group mean (middle horizontal line), overlap marks (upper and lower shorter horizontal lines), and 95% confidence interval (between upper and lower vertices).

Comparison of the mean age between the failure and success groups for the sitting position is shown in Fig 2A. The mean age was significantly higher in the failure group (74.0 ± 11.5 years) than in the success group (67.7 ± 12.4 years) (*P* = 0.0085; *t*-test). On the other hand, there were no statistically significant differences between the failure and success groups in mean duration of drop use (2.2 ± 3.9 vs. 3.9 ± 6.2 years respectively, *P* = 0.0915; *t*-test) and in mean corrected VA (0.83 ± 0.59 vs. 0.86 ±0.52 respectively, *P* = 0.8064; *t*-test). However, the mean spherical equivalent refraction was significantly higher in the failure group (-0.38 ± 2.37) than in the success group (-1.94 ± 3.41) (*P* = 0.0100; *t*-test).

Comparison of the mean age between the failure and success groups for the supine position is shown in Fig 2B. There was no statistically significant difference in the mean age (72.3 ± 12.9 vs. 70.1 ± 12.0 years, respectively, *P* = 0.3849; *t*-test) between the two groups. Similarly, no statistically significant differences were detected between the two groups in mean duration of drop use (2.6 ± 4.6 vs. 3.3 ± 5.5 years respectively, *P* = 0.5213; *t*-test), mean corrected VA (0.88 ± 0.63 vs. 0.83 ± 0.51, *P* = 0.6668; *t*-test), and mean spherical equivalent refraction (-0.50 ± 0.52 vs. -1.54 ± 0.37, *P* = 0.1059; *t*-test).

### Success rates of eye drop instillation by reason for hospitalization in sitting and supine positions

**Fig 3:**
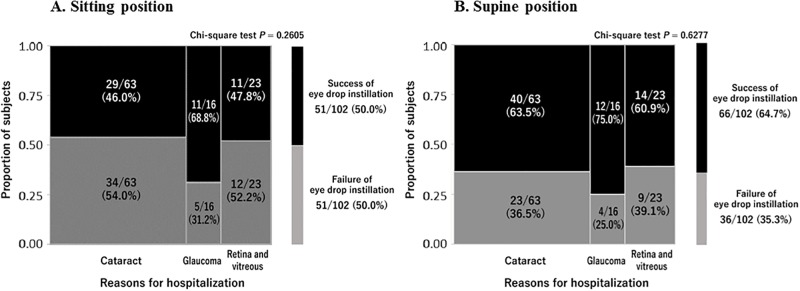
Success rates of eye drop instillation in sitting and supine positions by type of disease. Data are expressed as n (%).

The success rates of eye drop instillation in the sitting position by reason for hospitalization (type of disease) are shown in Fig 3A. Twenty-nine (46.0%) of 63 patients with cataract, 11 (68.8%) of 16 patients with glaucoma, and 11 (47.8%) of 23 patients with retinal and vitreous diseases successfully instilled the eye drop. There was no significant difference in success rate among the three disease types (*P* = 0.2605; chi-square test).

The success rates of eye drop instillation in the supine position by reason for hospitalization are shown in Fig 3B. Forty (63.5%) of 63 patients with cataract, 12 (75.0%) of 16 patients with glaucoma, and 14 (60.9%) of 23 patients with retinal and vitreous diseases successfully instilled the eye drop. There was also no significant difference in success rate among the three disease types (*P* = 0.6277; chi-square test).

### Success rates of eye drop instillation in sitting and supine positions between patients aged <75 and patients aged ≥75 years old

**Fig 4:**
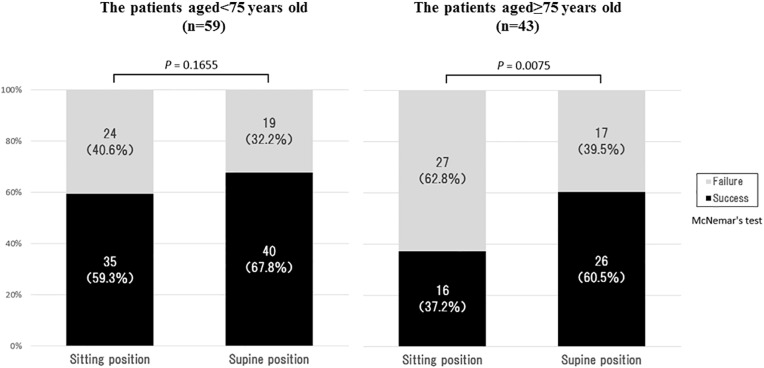
Comparison of success rate between patients aged <75 years and those aged ≥75 years. Data are expressed as n (%).

Comparison of the success rate of eye drop instillation between patients aged under 75 years and those above 75 years by instilling in the sitting and supine positions is shown in Fig 4. The success rates in the under 75 age group were 59.3% (35 of 59 patients) in the sitting position and 67.8% (40 of 59 patients) in the supine position, with no significant difference depending on the posture (*P* = 0.1655; McNemar’s test). Meanwhile, the success rates in the 75 years or older age group were 37.2% (16 of 43 patients) in the sitting position and 60.5% (26 of 43 patients) in the supine position, with a significantly higher success rate in the supine position compared to the sitting position (*P* = 0.0075; McNemar’s test).

## Discussion

Our results showed that the success rate of eye drop instillation was significantly higher when applied in the supine position than in the sitting position in a group of Japanese patients aged 70.2 ± 12.3 years with cataract, glaucoma, or retinal and vitreous diseases examined in the present study. The mean age was significantly higher in the eye drop failure group (74.0 ± 11.5 years) than in the eye drop success group (67.7 ± 12.4 years) when instilled in the sitting position, whereas no statistically significant difference in mean age was observed between the two groups in the supine position.

One of the effects of aging is a progressive reduction of physical function. Aging is associated with progressive physiologic changes that lead to deterioration of the structure and function of the musculoskeletal system [12–13], and this results in a decline in the functional performance of elderly people [14–15]. We speculate that elderly people may have difficulties in applying eye drops due to age-related postural changes such as kyphosis. Age-related kyphosis affects 20–40% of older adults and can be described as an exaggerated anterior curvature of the thoracic spine associated with aging [16].

Because of this physical characteristic, some elderly patients may have greater difficulties to turn the face upward in the sitting position. Furthermore, it is necessary to bend the neck backward firmly for accurate instillation. In the comparison of the success rate of eye drop instillation among patients aged over 75 years, significantly higher success rate was seen in the supine position compared to the sitting position. Conventionally, “elderly” has been defined as a chronological age of 65 years or older in many countries [17–19]. However, the ageing process is not uniform across the population due to differences in genetics, lifestyle, and overall health [20]. An analysis of the physical and psychological health of the elderly in Japan shows that deterioration of walking speed and grip strength have been delayed by 5–10 years among elderly people at present compared with 10–20 years ago [21]. Furthermore, the Joint Committee of the Japan Gerontological Society and the Japan Geriatrics Society recently proposed to define elderly as those aged over 75 years in Japan [22]. Therefore, “75 years of age” was deemed useful as the cut-off age for determining patient’s age-related physical ability in the present study.

There is a possibility that patients with ocular diseases have greater difficulties in eye drop instillation because they have very limited eyesight. Previous studies have suggested that factors associated with poor technique include poor manual dexterity, older age [4, 10], limited school education [4, 23], and poor vision [3, 23–24]. Hennessy et al. [10] reported that eye drop administration was a problem for patients with glaucoma and retinal diseases having impaired vision, suggesting the need for new therapeutic delivery systems. Low VA may influence eye drop instillation capability. However, in the present study, chi-square test detected no statistically significant difference in success rate among three disease types when instillation was done in both sitting or supine positions. Therefore, if our elderly patients have difficulties with eye drop instillation in the sitting position, it is appropriate to instruct them to instill in the supine position as one of the coping strategies.

In the present study, 30 subjects did not successfully instill the eye drops both in sitting and supine positions. Apart from fundamental problem with the technique of eye drop instillation, another possible reason for the failure may be due to the patients closing the eyelids by an instinct protective reflex. As a result, the eye drops fell on the cheeks or eyelids, or the tip of the nozzle touched the eyelids.

There were several limitations in the present study. First, we did not determine whether the intervention (eye drop instillation in the supine position) results only in short-term improvement. There may also be variability in performance. Therefore, a single demonstration may not be adequate to accurately assess technique. Second, we did not examine the success rate using various types of ophthalmic bottles, which may have influenced the results of statistical analysis. Third, all the subjects in the present study had poor visual acuity. Further study is required to examine the effect of body position on the success rate of eye drop instillation in subjects with good visual acuity. Despite these limitations, the present study identified possible physical factors that may impact patient’s performance, and this information would help patients to seek additional support in optimizing their technique. Eye drops are not always easy to administer compared to oral dosing. If elderly patients struggle to instill an eye drop properly even though they understand how to do it correctly, encouraging them to apply eye drops in the supine position could be one of technical strategies to reduce barriers to adherence.

Successful ophthalmic treatment depends on appropriate eye drop administration, and instructing proper application technique can remove potential barriers for achieving maximum therapeutic benefit. An important factor of poor adherence to ophthalmic treatment is incorrect dosing technique [25], and individual instructions that address the needs of elderly patients is also essential to prevent disease progression and sight loss. Even if we carefully give our patients technical medication instructions including the use of eye drop aids and instillation in the supine position, some patients may still experience difficulties in delivering eye drops on the ocular surface. In addition, according to a previous study of visually impaired patients with glaucoma or retinal diseases, 17% of patients relied on another person to administer the drops [10]. Healthcare professionals may need to offer enhanced support to patients by communicating closely with their families in order to reduce barriers to good compliance.

## Conclusion

Since revolutionary eye drop bottles and ophthalmic compliance aids have not been developed, patients including elderly persons should be recommended to apply eye drops in the supine position for improving adherence to ophthalmic treatment. Adherence may be further improved by mitigating the burden of eye drop administration through instillation in a supine position.
